# Supplementary material for: Development of A Standardized Opsonophagocytosis Killing Assay for Group B Streptococcus and Assessment in an Interlaboratory Study
Source: Vaccines (Basel). 2023 Nov 9;11(11):1703. doi: 10.3390/vaccines11111703 (PMC10675794; doi:10.3390/vaccines11111703)
Supplement: Supplementary file 1 [file vaccines-11-01703-s001.zip › vaccines-2597703-supplementary.pdf]

Supplementary materials

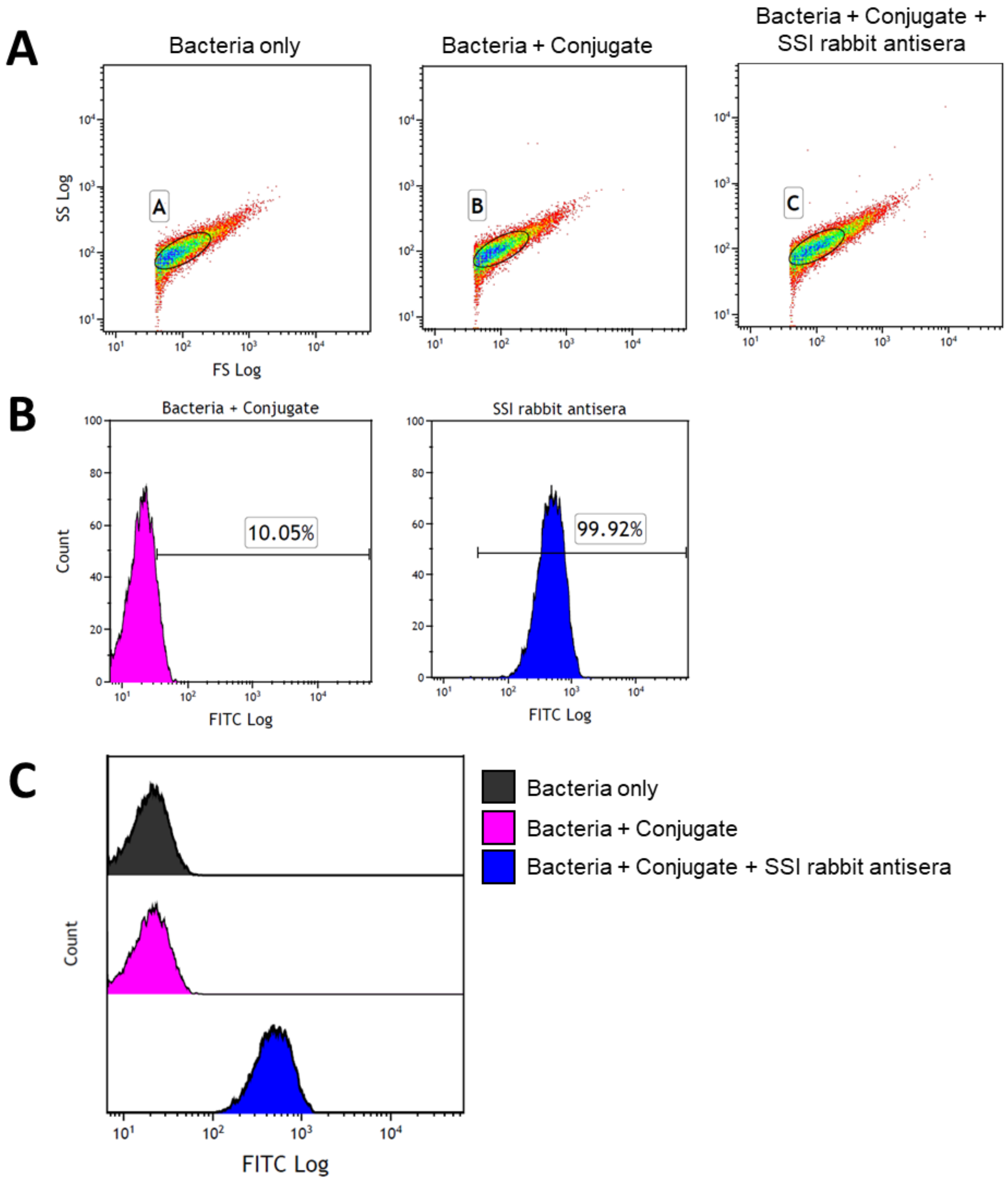

Figure S1. Binding of rabbit antisera to 49 GBS isolates by flow cytometry. (A) The main bacterial population was gated by forward scatter and side scatter. (B) Fluorescein isothiocyanate (FITC) histogram plot of negative control well (bacteria and conjugate) was used to setup gating strategy using a 10% lower limit. The same gate was applied to each isolate with SSI rabbit antisera. Mean fluorescence values for each isolate were multiplied by gated % of counts to obtain a mean fluorescence index (MFI) value. Each isolate was tested in duplicate: the mean and standard deviation were calculated. (C) Overlay plot of controls and test sample indicating fluorescence shift.

Serotype	Strain identifier	Mean fluorescence index (n = 2)					Standard deviation				
		SSI Ia	SSI Ib	SSI II	SSI III	SSI V	SSI Ia	SSI Ib	SSI II	SSI III	SSI V
	**PHE 1**	59520	20949	176	1393	2075	8495	696	52	68	22
	**PHE 2**	54280	41746	122	2616	4083	4548	611	84	20	109
	**PHE 3**	NT [#]	NT [#]	NT [#]	NT [#]	NT [#]	-	-	-	-	-
	**PHE 4**	NT [#]	NT [#]	NT [#]	NT [#]	NT [#]	-	-	-	-	-
	**PHE 5**	55003	22063	93	2077	5249	9852	1239	79	0	158
**Ia**	**PHE 6**	72793	11237	211	709	402	5602	216	27	114	44
	**PHE 7 ★**	59330	25826	119	1783	1242	5190	116	100	37	60
	**515**	58045	15782	76	857	2628	12250	730	109	60	158
	**NCTC 9993 (O90R)**	NT [♦]	NT [♦]	NT [♦]	NT [♦]	NT [♦]	-	-	-	-	-
	**NCTC 11078 (A909)**	43374	39205	940	3937	261	5396	90	48	627	131
	**NCTC 12906 (335)**	38050	16829	-557	851	3524	84	37	580	3	192
	**PHE 8**	NT [#]	NT [#]	NT [#]	NT [#]	NT [#]	-	-	-	-	-
	**PHE 9 ★**	74552	159318	306	2483	13594	3554	18012	202	245	5928
	**PHE 10**	7460	9506	92	5431	3959	1092	1887	2	2145	921
	**PHE 11**	NT [+]	NT [+]	NT [+]	NT [+]	NT [+]	-	-	-	-	-
**Ib**	**PHE 12**	25827	170360	116	2205	7809	5282	23807	162	118	1070
	**PHE 13**	33784	155255	586	5246	9704	3162	9818	418	1321	837
	**PHE 14**	22108	160605	373	1326	6636	4063	72738	155	783	114
	**H36B**	12496	61882	152	1907	704	232	5674	72	1176	171
	**Reference NCTC 8187**	15118	107760	148	1344	11627	4763	17124	180	609	1076
	**NCTC 8180 (S102)**	NT [+]	NT [+]	NT [+]	NT [+]	NT [+]	-	-	-	-	-
	**PHE 15**	10237	16004	40560	1388	910	1566	582	7135	4	92
	**PHE 16**	NT [+]	NT [+]	NT [+]	NT [+]	NT [+]	-	-	-	-	-
	**PHE 17**	7075	31	12484	5598	57	888	48	2466	574	94
	**PHE 18**	NT [♦]	NT [♦]	NT [♦]	NT [♦]	NT [♦]	-	-	-	-	-
**II**	**PHE 19 ★**	14199	178	27948	22809	910	561	193	6622	4359	466
	**PHE 20**	14947	15528	18485	3243	9360	461	740	1366	306	141
	**PHE 21**	13165	386	18671	18933	545	183	409	916	498	77
	**NCTC 11079 (18RS21)**	NT [+]	NT [+]	NT [+]	NT [+]	NT [+]	-	-	-	-	-
	**NCTC 8183 (S84)**	NT [+]	NT [+]	NT [+]	NT [+]	NT [+]	-	-	-	-	-
	**PHE 22 ★**	8629	60	-130	90338	851	3349	163	1	41735	55
	**PHE 23**	8878	118	-6	77455	685	778	106	8	38820	539
	**PHE 24**	NT [+]	NT [+]	NT [+]	NT [+]	NT [+]	-	-	-	-	-
	**PHE 25**	NT [+]	NT [+]	NT [+]	NT [+]	NT [+]	-	-	-	-	-
**III**	**PHE 26**	8888	94	14	111007	427	878	11	10	53649	79
	**PHE 27**	NT [+]	NT [+]	NT [+]	NT [+]	NT [+]	-	-	-	-	-
	**PHE 28**	NT [+]	NT [+]	NT [+]	NT [+]	NT [+]	-	-	-	-	-
	**COH-1**	NT [#]	NT [#]	NT [#]	NT [#]	NT [#]	-	-	-	-	-
	**NCTC 11080 (M216)**	10584	880	63	65630	541	1744	51	34	9145	189
	**NCTC 8184 (S122)**	NT [+]	NT [+]	NT [+]	NT [+]	NT [+]	-	-	-	-	-
	**PHE 29**	22248	23456	180	1552	119539	1929	7737	48	3	18219
	**PHE 30 ★**	9592	361	79	6944	22089	2976	240	52	286	6873
	**PHE 31**	NT [+]	NT [+]	NT [+]	NT [+]	NT [+]	-	-	-	-	-
	**PHE 32**	13284	881	23	9170	46516	309	713	5	2111	45167
**V**	**PHE 33**	16128	19635	134	2431	166674	2273	986	64	221	3540
	**PHE 34**	NT [#]	NT [#]	NT [#]	NT [#]	NT [#]	-	-	-	-	-
	**PHE 35**	NT [+]	NT [+]	NT [+]	NT [+]	NT [+]	-	-	-	-	-
	**CJB111**	NT [#]	NT [#]	NT [#]	NT [#]	NT [#]	-	-	-	-	-
	**NCTC 13946 (Prague 1984)**	NT [+]	NT [+]	NT [+]	NT [+]	NT [+]	-	-	-	-	-

Table S1. Mean fluorescence index values (n=2) and standard deviation of serotype-specific IgG to GBS isolate using rabbit antisera. Several isolates were excluded from further testing (not tested; NT) as denoted by NT [#] (no protein mAb binding), NT [+] (sensitive to complement and dHL60 and NT [♦] (extreme OPKA outlier). The isolate selected to represent each serotype (★) was accessioned at National Collection of Type Cultures (NCTC).
